# Imaging tools for plant nanobiotechnology

**DOI:** 10.3389/fgeed.2022.1029944

**Published:** 2022-12-08

**Authors:** Bin Zhao, Zhongxu Luo, Honglu Zhang, Huan Zhang

**Affiliations:** ^1^ School of Agriculture and Biology, Shanghai Jiao Tong University, Shanghai, China; ^2^ School of Biomedical Sciences and Engineering, South China University of Technology, Guangzhou International Campus, Guangzhou, China; ^3^ Department of Chemistry, College of Chemistry and Materials Science, Jinan University, Guangzhou, China

**Keywords:** plants, nanoparticles, imaging, nanobiotechnology, characterization

## Abstract

The successful application of nanobiotechnology in biomedicine has greatly changed the traditional way of diagnosis and treating of disease, and is promising for revolutionizing the traditional plant nanobiotechnology. Over the past few years, nanobiotechnology has increasingly expanded into plant research area. Nanomaterials can be designed as vectors for targeted delivery and controlled release of fertilizers, pesticides, herbicides, nucleotides, proteins, etc. Interestingly, nanomaterials with unique physical and chemical properties can directly affect plant growth and development; improve plant resistance to disease and stress; design as sensors in plant biology; and even be used for plant genetic engineering. Similarly, there have been concerns about the potential biological toxicity of nanomaterials. Selecting appropriate characterization methods will help understand how nanomaterials interact with plants and promote advances in plant nanobiotechnology. However, there are relatively few reviews of tools for characterizing nanomaterials in plant nanobiotechnology. In this review, we present relevant imaging tools that have been used in plant nanobiotechnology to monitor nanomaterial migration, interaction with and internalization into plants at three-dimensional lengths. Including: 1) Migration of nanomaterial into plant organs 2) Penetration of nanomaterial into plant tissues (iii)Internalization of nanomaterials by plant cells and interactions with plant subcellular structures. We compare the advantages and disadvantages of current characterization tools and propose future optimal characterization methods for plant nanobiotechnology.

## 1 Introduction

Plants constitute the most important element of global food security and carbon-oxygen balance. The domestication and cultivation of plants have fostered the growth of the population and the advancement of civilization, which was previously a tedious and time-consuming process. Nanobiotechnology has developed to advance a wide range of aspects of life science. As one of the most promising applications, plant nanobiotechnology is expected to effectively transform plants and optimize the plant culture system (Kah et al., 2019; Zhao et al., 2020). Nanobiotechnology has extended to diverse aspects of plant research, for example, using nanotechnology to achieve a more convenient and effective genetic modification of plants (Zhu et al., 2020); engineering nanomaterials as vectors for pesticides and fertilizer delivery to achieve efficient and environmentally friendly disease prevention and crop yield control (Zhao et al., 2017; Kah et al., 2018; Kumar et al., 2019; Lateef et al., 2019); to enhance plant stress and disease resistance in challenging environments (Cao et al., 2021; Chi et al., 2021); to intervene in plant growth and development (Verma et al., 2018; Priyanka et al., 2019); to directly engineer plants with special properties (Kwak et al., 2017; Lew et al., 2020a; Gordiichuk et al., 2021), etc. However, one of the questions that plant nanobiotechnology must address is how nanomaterials interact with plants. In the meantime, study on the location and transportation of nanomaterials in plants at various temporal and spatial scales is inevitable for understanding the interaction mechanism between nanomaterials and plants. Recent reviews (Wang et al., 2016; Hubbard et al., 2020; Chugh et al., 2021) have summarized the discoveries of the barriers that nanomaterials may encounter in plant cell penetration, and strategies to enhance the efficiency of engineered nanomaterials entry to plant cells. Multitemporal and spatial scale imaging techniques, that has been applied to animal systems (Sanchez-Cano et al., 2021), are successfully extended to plants (Wang et al., 2016). Currently developed imaging techniques have enabled real-time observations of the interactions between nanomaterials and individual plant cells or subcellular structures; surveillance of nanomaterial penetration and migration into plant organs; monitoring of the effects of nanomaterials on plant populations.

**FIGURE 1:**
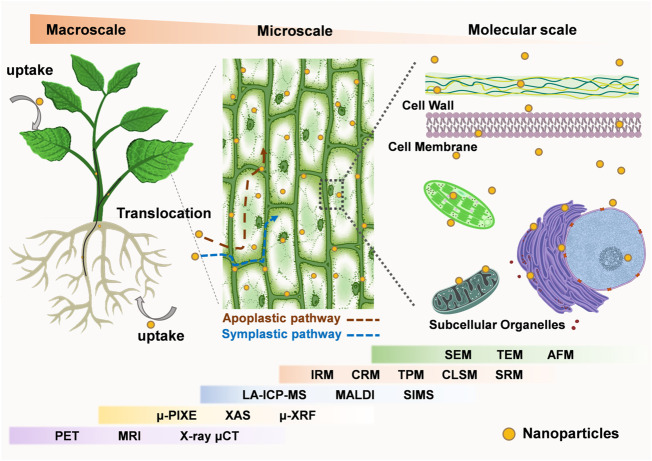
Imaging tools for plant nanobiotechnology at the micro-to-macro scale. At the microscopic scale, electron microscope (SEM, TEM, AFM) and optical microscope (SRM, CLSM, TPM, CRM, IRM), XAS, μ-XRF, SIMS can be used to observe the interaction of nanomaterials with plants at the cellular and subcellular levels. At the macroscopic scale, LA-ICP-MS, MALDI, μ-PIXE, PET, MRI, X-ray μCT are used to observe the distribution of nanomaterials in specific plant organs, tissues and the whole body. Meanwhile, some of the imaging techniques can be used for analysis across micro and macro scales. (Abbreviations: SEM, Scanning Electron Microscope; TEM, Transmission electron microscopy; AFM, Atomic Force Microscopy; SRM, Super-resolution microscopy; CLSM, Confocal Laser Scanning Microscope; TPM, Two-photon microscopy; CRM, Confocal Raman microscopy; IRM, Infrared spectral microscope; XAS, Synchrotron X-ray absorption spectroscopy; μ-XRF, Micro-X-ray fluorescence; SIMS, Secondary ion mass spectrometry; LA-ICP-MS, Laser ablation inductively coupled plasma mass spectrometry; MALDI, Matrix-assisted laser desorption ionization; μ-PIXE, micro-Particle-Induced X-Ray Emission; PET, positron emission tomography; MRI, magnetic resonance imaging; X-ray μCT, X-ray microcomputed tomography).

The aim of this review is to summarize the characterization tools in plant nanobiotechnology (Figure 1), which have been successfully used to analyze the interactions between nanomaterials and plants on a growing number of spatial scales, from micro to macro. Here, we present corresponding imaging tools to monitor nanomaterials-plant interactions on three length scales: 1) tracking the entry of nanomaterials into plant cells and their interactions with sub-organelles; 2) monitoring of nanomaterial penetration and migration in specific tissues; 3) surveillance of the migration and accumulation of nanomaterials between different organs in whole plants. By comparing the benefits and limits of different characterization techniques and proposing promising characterization tools for plant nanobiotechnology, we believe this review will benefit future researchers in choosing more appropriate tools in their research.

## 2 Tracking the entry of nanomaterials into plant cells and their interactions with sub-organelles

One of the most striking differences between plants and animals at the cellular lever is that plant cells have a rigid cell wall that surrounds the cell membrane, which is the first hurdle for nanoparticles entry. Moreover, the interaction of nanomaterials with sub-cellular plant cell structures is also an important factor for determination of the final fate of nanomaterials. Through rational design of nanomaterials, it is possible to enhance their internalization efficiency, thus, it is of great importance to study and observe the process of nanomaterials interacting and entering plant cells to facilitate design of more efficient transporters in plants.

### 2.1 Electron microscope

Electron microscopy is a widely used imaging tool with high resolution, and it has two main types: scanning electron microscope (SEM) and transmission electron microscope (TEM), both instruments are photographed *via* an electron beam produced by an electron cannon at a relatively high acceleration voltage. Therefore, EM can be used to image microscopic biological structures ranging from individual cells to biomolecules, like proteins, with sizes ranging from micrometer (µm) to nanometer (nm).

#### 2.1.1 Transmission electron microscope

TEM employs a high energy electron beam to image a thin sample *via* amplitude and phase variations of the transmitted beam. Owing to the small electron de broglie wavelength, TEM has an extremely high resolution (∼0.07 nm) (Ludwig, 2013; Falsafi et al., 2020), making it a powerful tool to observe fine microstructures. TEM equipped with an electron energy loss spectroscopy detector can perform elemental analysis of the sample, in addition, electron diffraction analysis of selected area can analyze the crystal structure of the sample. Till now, TEM have been widely used in physics, chemistry, material, and life sciences (Kociak and Zagonel, 2017; Zhang L. et al., 2020; Hage et al., 2020). In animal system, TEM has been used extensively to study the morphology of cells and their organelles, investigating the interactions between nanomaterials and mammalian cells, which makes the transition to study the interaction between nanomaterials and plant cells more adaptable.

TEM was utilized to study the uptake, translocation, and transmission of carbon nanomaterials in rice plants (Lin et al., 2009). Researchers observed a large number of fullerene C_70_ aggregates in the vacuoles of 2-week-old rice plant leaf cells, providing a direct evidence that C_70_ can be transported from the plant roots to the leaves. It suggested that C_70_ may enter the roots of the plant by osmotic pressure and capillary force and then be transported into the foliar cells. To study the toxicity mechanism of nickel oxide nanoparticles (NiO-NPs) to tomatoes, Faisal et al. (2013) observed tomato root cells using TEM, and found that NiO-NPs not only presented in external regions of parenchymal confluence cells, but also translocated in the cytoplasm of cells and aggregated around vacuoles. Besides, increased number of peroxisome and mitochondrial, degeneration of crystals and condensed nuclei were also observed in NiO-NPs treated root cells. The study pointed out that the dissolution of Ni ions from NiO-NPs can induce cell death by triggering the mitochondrial dependent intrinsic apoptotic pathway. Using TEM observation and further analysis by energy dispersive spectroscopy (EDS), researchers discovered that molybdenum-sulfur (Mo-S) crystals can be self-assembled in plant cells (Chi et al., 2021) by spraying MoCl_5_ and cysteine (with 200 mM salt for 7 days) on the leaves, and demonstrated that the intracellular crystals can augment the photosynthesis and improve plant stress resistance through multiple mechanisms, such as promoting Ca^2+^ signal transduction, removal of reactive oxygen species (ROS) etc.

**FIGURE 2:**
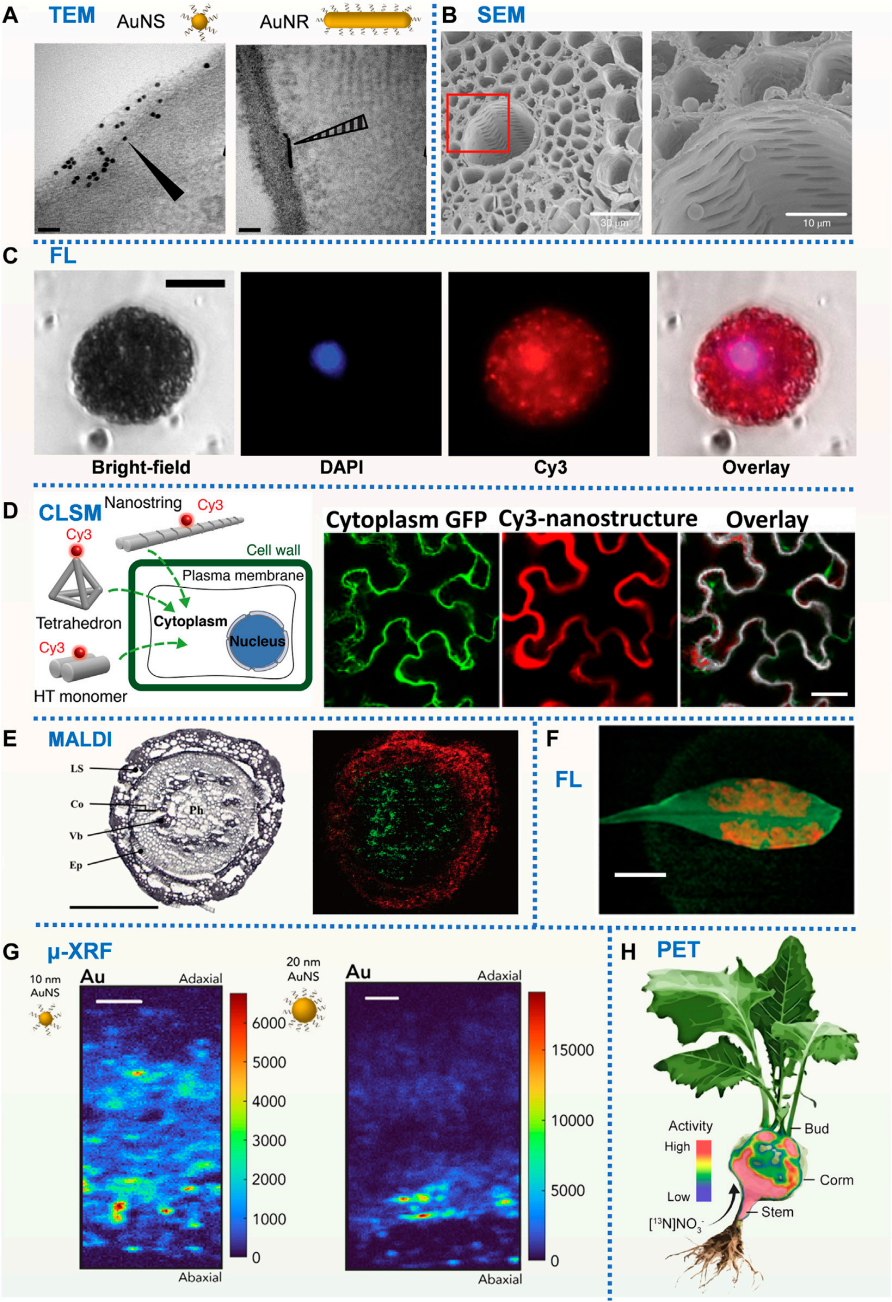
**(A)** TEM images of *N. benthamiana* plants 24 h post-infiltration with DNA-functionalized AuNS and AuNR. Scale bars, 50 nm (Zhang et al., 2022b). **(B)** SEM images of PS microbeads in the xylem vessels and cortical tissue. Scale bars, 30 µm (left) and10 µm (right) (Li et al., 2020). **(C)** Fluorescence microscopy images of Cy3-DNA-CNTs internalization into isolated protoplast cytosol and nucleus after incubation with DAPI-stained protoplasts. Scale bars, 20 µm (Demirer et al., 2019c). **(D)** DNA nanostructure internalization into and colocalization with mGFP5 *Nb* cytoplasm using confocal microscopy. Scale bars, 40 µm (Zhang et al., 2019). **(E)** MS imaging of a wheat crown at the seedling stage. Polyphenol glycoside (red) and phosphatidylcholine (green). Scale bar, 500 µm (Bhandari et al., 2015). **(F)** Fluorescence macroscopy images of DNA-wrapped SWNTs within the leaf lamina of *A. thaliana*. Scale bar, 1 cm (Giraldo et al., 2014). **(G)** μ-XRF Au distribution map of *N. benthamiana* leaf cross-section exposed to 5 μg of 10 and 20 nm DNA-AuNSs. Scale bars, 50 μm (Zhang et al., 2022b). **(H)** Positron imaging of plant nitrogen dynamics (Liang et al., 2011).

A library of gold nanomaterials (AuNPs) with different sizes and morphologies were employed to investigate the impact of nanomaterial morphology on its interaction with plant tissues and cells (Zhang et al., 2022a). Subcellular localizations of AuNPs were tracked by TEM (Figure 2a), and spherical AuNPs of all sizes were found to be uniformly distributed at the periphery of the cell wall/membrane and could not enter the plant cell. However, rod-shaped AuNPs were found inside plant leaf cells and have different interacting angles with the cell wall. By statistically analyzing the interaction angles of rod-shaped AuNPs with the cell wall, they concluded that rod-shaped AuNPs might enter plant cells using a rotation (from parallel to vertical) based way. By employing AuNPs as carriers for siRNA delivery, this study shows that no internalization of nanocarriers is required for efficient RNA delivery in plants.

TEM imaging is performed by collecting electrons through the sample, which provides information about the morphology and structure of the sample. TEM can facilitate the study of the localization and interaction processes of nanomaterials with plant cells and subcellular structures, which can help to better understand the kinetic processes of nanomaterials with different physical properties interaction with plant cells.

#### 2.1.2 Scanning electron microscopy

SEM uses electrons for imaging by scanning the surface with a low-energy beam (typically from 1 to 30 keV) with a resolution up to nm, and collects information of morphology and composition of the sample. SEM equipped with an energy dispersive X-ray spectrometer (EDX) can estimate the composition and structure of the sample. SEM has been widely used in material and life sciences, especially in several fields of biological science such as investigating cell morphology, bacteria, and their interactions with cells etc. (Titze and Genoud, 2016; Joseph et al., 2017; Russell et al., 2017; Kalsoom et al., 2018).

To investigate whether microplastics will be taken up by crops (wheat and lettuce) growing in hydroponic systems or a sandy soil, the distribution of sub-micron polystyrene (PS) microspheres and microns in the roots, stems and leaves of crops were characterized by SEM (Li et al., 2020, Figure 2B). Submicron and micron PS was found to penetrate the crop stele by crack entry patterns at the lateral root initiation site. Further study verified that the movement mediated by transpiration pull promoted the uptake of microplastic particles by plants. The work pointed out the microplastic waste must be given urgent consideration in management of global plastic pollution since microplastics often presented in wastewaters employed for agricultural irrigation. CuO and Al_2_O_3_-NPs on tomato root surface were characterized using SEM equipped with EDX to evaluate the toxicity of metal oxide nanopollutants (Ahmed et al., 2018). Internalization and translocation of the NPs were further confirmed *via* TEM and ICP-MS. Further investigation revealed that metal oxide nanopolutants can lead to depression in tomato growth, dry biomass, and total soluble protein content, and was harmful to photosynthesis. Cultured carbon nanofiber-loaded copper nanoparticles (Cu-CNFs) with *Cicer arietinum* seeds to investigate their effects on the germination and following plant growth. SEM imaging and EDX based element mapping demonstrated that there were fibers in the shoot and the copper content in the roots increased, indicating that Cu-CNFs can migrate in plants and release micronutrient (Cu) to promote plant growth. They concluded that Cu-CNFs can serve as an efficient growth stimulant for crops, and nano-sized Cu-CNFs have the potential to be nanofertilizers on agricultural land. SEM provides 3D imaging of the sample surface, thus it is more suitable for studying the interactions of nanomaterials with plant cell walls or plant tissue surfaces.

Despite all the above mentioned applications, there are still some limitations of EM when used for tracking nanoparticles and study nanomaterial-plant cell interactions. One of the most important challenges is sample preparation, which is complicated, labor consuming and has the risk to damage or contaminate the sample. The morphology of the treated samples is often altered and thus cannot accurately reflect the initial state of nanomaterial-plant interactions. Moreover, the EM operating environment is strict, making EM imaging cost high. Moreover, EM is not suitable for *in vivo* or *in situ* imaging.

### 2.2 Fluorescence microscopy

Fluorescence microscopy is the most used methods for imaging in both animal and plant system since it offers the benefits of simple operation, low cost, high sensitivity and multi-channel imaging. The representative laser scanning microscopy is one of the most important advances in the field of fluorescence microscopy in recent decades (Tata and Raj, 1998; Amos and White, 2003; Halbhuber and König, 2003). It has been widely applied in biological research due to the point-by-point scan of sample and compatibility with 3D real-time living cell imaging, through which the dynamic molecular process and interaction of nanoparticles with cells can be easily monitored.

#### 2.2.1 Confocal laser scanning microscopy

In the past, CLSM has been heavily relied upon for plant nanobiotechnology due to its high compatibility with various samples. There are plenty of reviews summarizing the nanoparticle-mediated gene delivery in plants, in which CLSM was a commonly used method for characterization (Demirer and Landry, 2017; Chuah and Numata, 2018; Demirer et al., 2019a; Demirer et al., 2019b; Demirer et al., 2020; Dong and Ronald, 2021; Yan et al., 2022). Here, we are briefly reviewing the most recent study done in the past five years and how CLSM contributed to their significant findings.

DNA nanostructures with controllable size, shape, stiffness, and compactness were designed, labelled with Cy3 fluorophore and infiltrated through the abaxial surface of leaves to assess their cellular uptake in mGFP5 *N. benthamiana* (*Nb*) transgenic plants *via* CLSM (Figure 2D). By simultaneously imaging the Cy3 fluorescence of nanostructures and the intrinsic cytoplasmic GFP fluorescence produced by plant cells, they provided an index to assess the relative internalization efficiency of different nanostructures into plant cells (Zhang et al., 2019; Zhang H. et al., 2020). Co-localization fraction of Cy3 fluorescence (nanostructures) with GFP fluorescence (plant cell cytoplasm) was used to determine the extent of nanostructure internalization into the cell cytoplasm. Results showed that the size and stiffness were two key factors that determined the internalization efficiency of DNA nanostructures. Then, they employed the colocalization strategy based on CLSM to study the internalization of polyethyleneimine-functionalized gold nanoclusters (PEI-AuNCs) to mature plants, and discovered that PEI-AuNCs can enter plant cells quickly (∼0.5 h), and only when loaded on AuNCs, single-stranded DNA and RNA can pass through the cell wall and cell membrane and finally enter cells. These findings suggested that gold nanoclusters could be used as a siRNA carrier (Zhang et al., 2021). Recently, they utilized CLSM to monitor the capacity of gold nanoparticles (AuNPs) of different sizes and morphologies to transport in the leaf tissue and to associate with plant cell walls. Cy3 signals from Cy3-DNA-AuNPs and GFP signals from the transgenic *Nb* cytosol (as marker) were captured by CLSM, the colocalization fraction between Cy3 and GFP channels reflected the ability of Cy3-DNA-AuNPs to diffuse within the leaf interstitial space and associate with (and/or enter) plant cells. Statistical results at different incubation time points showed that DNA-AuNRs with diameters less than 15 nm can diffuse within the leaf space and bind to plant cell walls in a size- and shape-dependent manner while the diffusion of 20 nm AuNPs got limited in between cells (Zhang et al., 2022a).

To confirm the interiorization of carbon nanotube (CNTs) within mature leaf cells, labelled the CNTs with a Cy3-DNA and infiltrated it to foliar cells that continuously expressed GFP. CLSM results showed that Cy3-DNA alone did not show co-location of Cy3 fluorescence with GFP, while Cy3-DNA labelled CNTs observed 62% co-location between Cy3 and the intracellular channels of GFP. The results indicated that Cy3-DNA alone cannot be internalized into cells, while CNTs can be efficiently internalized into plant cytoplasm and nucleus (Demirer et al., 2019b, Figure 2C).

Apart from the size and morphology, Hu et al. (2020) found that surface charge, hydrophobicity and surface tension in nanomaterials were also important parameters affecting cell internalization and subcellular targeting in nanomaterials using CLSM mediated study. Modification of nanomaterials with specific sequences of peptides can enable targeted gene delivery to different organelles. Keiji Numata group employed CLSM to co-localize the autofluorescence of plant chloroplasts (or MitoTracker Red CMXRos-labeled mitochondrial) with fluorescence of nanomaterials to quantify the efficiency of nanomaterials entering into target plant cell chloroplasts or mitochondria (Miyamoto et al., 2018, 2021; Yoshizumi et al., 2018; Thagun et al., 2019; Law et al., 2022). Quantum dots (QDs) modified by rational designed polypeptide, and CLSM was employed to verify that the modified QDs can achieve effective subcellular organelle (chloroplast) targeting and can manipulate chloroplast function and redox state *in vivo* (Santana et al., 2020).

To summarize, CLSM allows nanomaterials to co-localize with plant cells or subcellular structures *in vivo* and is thus frequently used to assess the relative efficiency of nanomaterials to enter plant cells or target subcellular structures. Despite the advantages, CLSM has its limitations, such as the need for fluorescent labeling of nanomaterials or plant cells. Confocal Raman Spectroscopy (CRM), which combines CLSM technology with laser Raman spectroscopy technology, can serve as an alternative imaging tool for non-destructive, chemically selective, label-free, qualitative and quantitative analysis of cells and nanomaterials (Schenzel et al., 2005; Luis et al., 2014). Several reports have shown the use of CRM for imaging mammalian cells and visualizing the uptake of drug vectors and nanomaterials (Kann et al., 2015). Currently, the application of CRM in plants focuses mainly on analysis of plant cell wall, regarding to molecular composition, structure and spatial arrangement (He et al., 2020). We believe CRM could be employed as a promising tool in plant nanotechnology, especially in analyzing interaction process of nanomaterial with plant cell wall Figure 1.

#### 2.2.2 Two-photon microscopy

Two-photon microscopy is another variation of a microscope that combines laser scanning and two-photon excitation. It excites fluorescent molecules by replacing one short-wavelength photon with two long-wavelength photons, thus can reduce phototoxicity and achieve better penetration depth with 3D resolution (Peter et al., 2000; Helmchen and Denk, 2005). Since two-photon excitation occurs only at the focal point of the lens, it is not necessary to have a confocal pinhole to isolate the background signal, thereby improving the detection efficiency. Despite these advantages, there are not much studies using two-photon fluorescence microscopy in plants Figure 2.

In order to determine the mechanism of copper oxide nanoparticle uptake in sweet potato roots, Bonilla-Bird et al. (2018) found that lignin kept CuO NPs at the outermost periderm of the tomato root, preventing CuO NPs from entering the inner tissue of the root through two-photon microscopy imaging, suggesting that CuO NPs are promising candidates for preservation of sweet potato roots without overloading the copper content. Two-photon microscopy was also employed to study the uptake of multiwalled carbon nanotubes (MWCNTs), TiO_2_, and CeO_2_ NPs in wheat roots (Wild and Jones, 2009), results showed that some MWCNTs penetrated the plant cell wall and entered the cytoplasm, while TiO_2_ and CeO_2_ NPs only aggregated on the surface of roots. Two-photon microscopy uses longer wavelengths for fluorescence excitation, resulting in low phototoxicity and high penetration depth, allowing for the observation of nanomaterial co-localization with deep plant tissues, which is very useful for studying plant uptake and deep tissue diffusion processes of nanomaterials.

#### 2.2.3 Super-resolution microscopy

As we know, conventional optical microscopes have a resolution limit of ∼200 nm due to the diffraction limit of light, which restricts their application to study structures below that limit. In principle, SPM overcomes the optical diffraction restriction and bridges the resolution gap between optical and electron microscopy together with the advantages of simple sample preparation, higher resolution and *in vivo* imaging. SPM is a quite promising tool for bioscience. The recently developed SPM techniques, such as structured illumination microscopy (SIM), photoactivation localization microscopy (PALM), stochastic optical reconstruction microscopy (STORM), and stimulated emission depletion microscopy (STED), have shown an increasing application in cell biology (Tan et al., 2018; Schermelleh et al., 2019; Jacquemet et al., 2020). Plant cell imaging *via* SPM is particularly challenging due to the optical heterogeneity of plant cells and varying refractive indices of plant cell organelles. Even though, the applications in this field are increasing recently, especially SIM, since it downgrades high-frequency information in the sample to the actual bandpass of the microscope and making the sampled high-frequency components visible. There are several reviews that summarized the progress of SPM application in plant cell imaging (Gutierrez et al., 2010; Komis et al., 2015b; Rillig et al., 2017), here we are listing some representative work illustrating how SPM can help study the subcellular organelles in plants, and how SPM can enlighten prospective researchers in plant nanobiotechnology (Gutierrez et al., 2010; Komis et al., 2015b; Ovečka et al., 2022).

STED has so far been successfully exploited to image proteins in plasma membrane nanodomains and obtained more accurate size (Demir et al., 2013). STED imaging of Arabidopsis cross-interference process showed that synaptonemal complex, a structure that zipped homologous chromosomes during meiosis, was essential in this process (Capilla-Pérez et al., 2021). Furthermore, a user-friendly and easy-to-follow protocol for super-resolution imaging of cortical microtubules in living cotyledons, petioles, and root cells using SIM to achieve sub-diffraction resolution (106 nm) was successfully developed (Komis et al., 2014; Komis et al., 2017). At the same time, the method enabled the monitoring of dynamic events in microtubule cortical arrays, including tracking the growth and contraction of individual microtubules and the branching and formation of new microtubules (Komis et al., 2015a). Fast super-resolution imaging of endosomes in the process of growing plant roots by SIM, and found that initial endosomes were dominated by continuous movement, and late endosomes were dominated by intermittent movement (Von Wangenheim et al., 2016). SIM was applied to explore the dynamic behaviors of cellulose synthase (CESA) particles in living plant cells (Duncombe et al., 2022). They improved the resolution by two-fold over confocal microscopy, and revealed that *Arabidopsis thaliana* CESA particles were more than twice as dense as previously estimated in the plasma membrane. SIM imaging method listed here have the great potential to be extended to study the dynamic interaction process between nanomaterials and plant cells and subcellular organelles *in vivo*. SRM overcomes the limits of optical diffraction and can achieve higher resolution, which can be used to observe the dynamic processes of nanomaterials interacting with plant subcellular structures or biomolecules, allowing for a better understanding of the mechanisms of nanomaterials entering or interacting with plant subcellular structures at molecular level.

In the aforementioned, fluorescence microscopy is an effective tool to monitor nanoparticle transport processes in plants, meanwhile, it is worth noting that some nanomaterials can be designed to detect specific molecules in plants *via* relative fluorescence intensity change or wavelength shift. Michael S. Strano Lab has developed a variety of *in vivo* molecular probes in plants based on the corona phase molecular recognition (CoPhMoRe) technique. For example, near-infrared fluorescence of DAP-dex (3,4-diaminophenyl-functionalized dextran) coated single-walled carbon nanotubes (SWNTs) can be selectively bleached by NO, and have been developed for *in vivo* NO detection in plants (Giraldo et al., 2014, Figure 2F). Similarly, ss (GT)_15_ oligonucleotides-encapsulated SWCNTs were designed for *in vivo* H_2_O_2_ detection in plant leaf (Lew et al., 2020b; Wu et al., 2020). This technique has also been used in plants for *in vivo* polyphenol (Nissler et al., 2022), nitroaromatic compounds (Wong et al., 2017), and Arsenite (Lew et al., 2021) detection in plants. Other nanomaterials, such as luminescent lanthanide metal-organic frameworks (Ln-MOFs), were developed for the detection of metal ions (Ag^+^, Cd^2+^, Fe^3+^, Cu^2+^) in plants (Liang et al., 2020). Recently, thiol-conjugated glucose-DNA aptamer sensor was designed for glucose detection through fluorescence resonance energy transfer (FRET) in plants (Mou et al., 2022).

As discussed above, fluorescent microscopy has outstanding advantages such as simple sample preparation, minimal sample damage, and *in vivo* multi-dimensional imaging with various and spatiotemporal resolution. Although widely used, fluorescence microscopes are also subjected to inevitable problems, such as phototoxicity, photobleaching, relatively low time resolution and low penetrating ability. Besides, nonfluorescent nanomaterials must be labelled for imaging, which might affect their surface properties and introduce unpredictable contaminants.

### 2.3 Mass spectrometry imaging

The mass spectrometric imaging (MSI) technique utilizes mass spectrometry to visualize the spatial distribution of compounds (such as elements, metabolites, peptides, or proteins, etc.) based on the molecular mass. MSI method uses ionization sources (such as ion beams, plasma beams, lasers, solvent sprays etc.) to first ionize the sample, and then ions were visualized through an analyzer. MSI involves techniques such as laser ablation inductive coupled plasma mass spectrometry (LA-ICP-MS), secondary ion mass spectrometry (SIMS), matrix-aided laser desorption/ionization (MALDI) and ionization by desorption electrolysis (DESI) spraying. Through collecting the point-by-point signal and selecting the corresponding element/compound peak, we can determine both the molecular compositions and spatial element distributions without labeling. There are several reviews (Boughton et al., 2016; Buchberger et al., 2018) introducing the application of MSI in plants, here we are focusing on its application in nanoparticle plant interaction analysis.

#### 2.3.1 LA-ICP-MS

LA-ICP-MS uses a laser beam to generate fine particles *via* laser ablation, and the ablated particles were ionized and analyzed by a mass spectrometer and superimposed to obtain an image of the elemental distribution of the sample. Currently developed femtosecond laser pulses have significantly improved the precision of LA-ICP-MS as well as the sensitivity. LA-ICP-MS can be used as an effective tool for studying the internalization and migration of nanomaterials in plants.

LA-ICP-MS was employed to study the distribution of ^107^Ag, ^55^Cu, ^63^Mn in soybean leaves cultured with silver nanoparticles (AgNPs) or silver nitrate (AgNO_3_). Results revealed a slight transfer of Ag from soil to leaves, regardless of the type of silver source (Chacón-Madrid and Arruda, 2018). However, when mapping the distribution of Cu and Mn elements, the presence of AgNO_3_ was found to seriously affect the dynamic equilibrium of Cu and Mn, since their leaf distribution was quite different from control plants and even plants processed with AgNPs. To investigate the uptake, transformation, transport, and deposition of La_2_O_3_ nanoparticles (NPs) to stem and leaves of *Pfaffa glomerata*, Neves et al. (2019) cultured *Pfaffa glomerata* plants with 400 mg/L of La_2_O_3_ NPs, then characterized by LA-ICP-MS and micro X-ray fluorescence (μ-XRF). Results showed that higher concentrations of La were observed in the main veins, and there were differences between the bulk La_2_O_3_ and La_2_O_3_ NPs groups. Besides, they found that LA-ICP-MS was more sensitive than μ-XRF and allowed better detection and visualization of La distribution in the leaf. To study the uptake, bioaccumulation, transfer, and localization of cerium oxide nanoparticles (CeO_2_ NPs) in radish (Raphanus sativus L.), The spatial distribution of CeO_2_ in edible roots was investigated by LA-ICP-MS imaging after culturing radish with CeO_2_ NPs (Wojcieszek et al., 2019). Results showed that the majority of cerium remains in roots with a low transportation up to leaves and stems, but after accumulation, they have the capacity to enter and migrate into the root tissue of the radish, which could raise food safety concerns.

#### 2.3.2 SIMS

Different from LA-ICP-MS, SIMS uses a high-energy paused primary ion beam to bombard the sample surface, atoms, or clusters of atoms of the sample to produce secondary particles, and these charged particles are tested by a mass spectrometer to obtain information of the sample. High-resolution secondary ion mass spectrometry (NanoSIMS) is currently capable of imaging most elements with high lateral resolution (down to 50 nm) and sensitivity. To study the effect of nanosized molybdenum octahedral (Mo_6_) clusters on the growth of rapeseed plants. Root incubated with Mo_6_ were analyzed by NanoSIMS (Aubert et al., 2012). Results revealed that nanoscale Mo_6_ were abundantly present in apoplasts and symplasts in the endoderm (cortex) and midcolumn, with a gradually decreasing concentration gradient from the outside to the inside. Interaction of surface-functionalized silver nanoparticles (AgNPs) with the green algae *Raphidocelis subcapitata* was investigated using a multimodal imaging approach (Sekine et al., 2017). Positively charged branched polyethyleneimine (bPEI) and negatively charged tannic acid (TA) were modified onto the surface of 10 nm and 60 nm AgNPs to obtain TA-AgNPs and bPEI-AgNPs for further study. Analyzed with NanoSIMS, Ag was found in algae exposed to 10 nm bPEI-AgNPs and aggregated outside the periplasmic space, whereas TA-AgNPs (10 and 60 nm) were only present on the cell surface, indicating that the internalization of AgNPs may depend on the surface function. Moore et al. (2012) used NanoSIMS to examine algae exposed to AgNPs and observed an overlap between ^32^S^−^ and ^107^Ag^+^ in the cell wall and cytoplasm, which indicated the co-accumulation of Ag and S in algal cells and indirectly confirmed the existence of Ag-sulfur complexes in algal cells.

#### 2.3.3 MALDI

Although LA-ICP-MS and SIMS can be used to map the element distribution of the nanoparticles, they are not suitable for the analysis of nanomaterials (for example, carbon nanomaterials, DNA nanomaterials, high molecular polymers, liposomes, etc.) which contain elements (such as C, H, O, N, P) that can be found in plants. MALDI is a soft ionization technique that utilizes a matrix to enable proper ionization of the molecules of interest, and the indistinguishable analytes using traditional ionization methods are extracted and co-crystallized with the matrix for further MS analysis. MALDI have been proved to be a powerful tool to delineate the spatial distribution of metabolites (Sturtevant et al., 2016; Bhandari et al., 2015, Figure 2E), alkaloids (Wu et al., 2007; John et al., 2018), lipids (Li et al., 2018; Qin et al., 2018; Liu B. et al., 2021) , macromolecules (Stahl et al., 1997) and proteins (Liu H. et al., 2021) in plants. For example, Shiono et al. (2017) imaged the phytohormone [cytokinein (tZ) and abscisic acid (ABA)] 2000 μg /mL in rice roots by Matrix-assisted laser desorption ionization time-of-flight mass spectrometry (MALDI−TOF−MS). Results showed that tZ was widely distributed about 40 mm posterior to the root apex, but was almost absent at the root apex, while ABA was mainly distributed at the root apex. This case demonstrated the potential ability of MALDI-TOF-MS to enable visualization of phytohormone localization as well as quantification. So far, there are no literature report of application of MALDI in nanomaterial related plant nanobiotechnology. However, we believe the technique especially MALDI−TOF−MS can be extended to analyze the distribution of nanomaterials in plants by identifying their characteristic peaks in mass spectrometry.

Mass spectrometry imaging can visualize the distribution of nanomaterials in plant tissues by a label-free method, and notably, it can also simultaneously image biomolecules such as biomarkers, metabolites, peptides or proteins, thus providing a clear advantage when studying the interaction of nanomaterials with biomolecules in plant tissues.

### 2.4 Synchrotron radiation techniques

SR is a technology that accelerates charged particles (such as electrons) at a velocity of near light to obtain an intense beams of photons with energies ranging from infrared to X-rays for imaging (Vijayan et al., 2015). Compared with other imaging techniques, SR technique has great advantages in providing both information with various spatial resolution (from organ or tissue level to cellular and subcellular level), distribution and chemical state in the meantime. There is a review by Wang, (2015) introducing the application of SR techniques in animals, environmental, soil and plants. In plant study, SR technique can be used to investigate metals and metalloids distributions in plants with benefits including primarily femtosecond sensitivity and micro-nano level spatial resolution as well as providing the valence status of elements. Current imaging modalities based on SR techniques include primarily micro-X-Ray Fluorescence (μ-XRF), synchrotron X-ray absorption spectroscopy (XAS) and micro-Particle-Induced X-Ray Emission (μ-PIXE).

#### 2.4.1 Micro-X-ray fluorescence and synchrotron X-ray absorption spectroscopy

μ-XRF can determine the distribution of various elements with high spatial resolution. XAS is commonly used to determine the chemical information of elements, including oxidation status, interatomic distance, coordination number, and speciation. Hence, by combining μ-XRF with XAS, the elementary distribution, composition and chemical speciation of the sample can be obtained simultaneously.

Elemental metal nanoparticles (Zhang et al., 2022b, Figure 2G) and Metal oxide (such as CuO, ZnO, CeO_2_, TiO_2_) NPs are the most suitable system to study *via* μ-XRF and XAS. Hernandez-Viezcas et al. cultured soybeans supplemented with 500 mg/kg ZnO NPs or 1,000 mg/kg CeO_2_ NPs in soil, then harvested soybean tissues (including pods) were subjected to μ-XRF and XAS analysis to determine the distribution and chemical information of Ce and Zn in NPs-treated plants. XAS results showed that soybean plants grown in soil soaked with ZnO NPs did not accumulate these NPs in seeds, and ZnO NPs can be decomposed in plants and exist as Zn-citrate complexes in seeds. However, Ce exists mainly in the form of CeO_2_ NPs in plants, and a small fraction of Ce (IV, the oxidation state of Ce in CeO_2_ NPs) is biotransformed to Ce (III). Consequently, CeO_2_ can be absorbed into food crops and accumulated in seeds, meaning that CeO_2_ NPs can reach the food chain and the next generation of soybean plants (Hernandez-Viezcas et al., 2013). Similarly, combined XAS and μ-XRF to analyze the distribution and chemical information of Zn in maize seedlings exposed to ZnO NPs. Results showed that most of the Zn absorbed by plants came from Zn^2+^ released by ZnO NPs, and accumulated in the form of zinc phosphate. ZnO NPs mainly accumulated in the epidermis, while a small part of ZnO NPs entered the cortex and root tip cells. Some ZnO NPs further entered the vascular system through the primary root-lateral root junction. However, no NPs were observed in shoots, which may be the decomposition and transformation of ZnO NPs in plants. To study the biological and toxicological effects of CeO_2_ and La_2_O_3_ NPs, Ma et al. (2015) characterized the distribution and chemical state of Ce and La in cucumbers treated with CeO_2_ and La_2_O_3_ NPs. μ-XRF and XAS analysis results showed that La mainly existed as phosphate or carboxyl complexes, while Ce mainly existed as CeO_2_ NPs, and only a small part of CeO_2_ was converted into Ce (III)-carboxyl complexes. The results indicated that La_2_O_3_ was absorbed by plants in the form of ions, whereas CeO_2_ penetrated into plants primarily in the form of CeO_2_ NPs. μ-XRF and XAS were employed to study the translocation and biotransformation of CuO NPs in rice roots exposed to NPs for 14 days (Peng et al., 2015). Researcher found that CuO NPs were able to enter the root epidermis, exodermis and cortex, and finally reached the endodermis. Meanwhile, the formation of lateral roots provided a potential route for NPs to enter the stele. Furthermore, the X-ray absorption near-edge structure (XANES) spectroscopy data showed that CuO NPs could be transported from roots to leaves, where Cu (II) was bound to cysteine, citrate and phosphate ligands, and even reduced to Cu (I).

#### 2.4.2 Micro-particle-induced X-ray emission

μ-PIXE is a non-destructive technique that could determines element distribution as well as quantitative analysis, which utilizes element-specific X-ray emission for imaging by exciting the sample with a highly focused beam of high-energy particles (usually protons). The use of a focusing microscope enables the μ-PIXE to have higher resolution as well as the capability of 3D imaging. To understand the migration and accumulation process of TiO_2_-NPs of different sizes and crystalline phases in wheat, plants were soaked in isopentane at -160 °C, then immediately embedded, sliced and freeze dried the samples for μ-PIXE analysis (Larue et al., 2012). Results showed that TiO_2_ NPs with a size of less than 36 nm accumulated in the roots and migrated into the whole plant tissue without dissolution or crystal phase change. However, TiO_2_ NPs with a size larger than 36 nm only accumulated in the parenchyma of wheat roots, and did not be transferred to the shoot. TiO_2_-NPs over 140 nm could not be absorbed by the wheat roots. Lettuce seedlings exposed to TiO_2_ and AgNPs were analyzed by μ-PIXE (Larue et al., 2016). Results indicated that AgNPs were more effectively absorbed by the root system than TiO_2_ NPs, meanwhile, Ag and Ti were observed in the stems, suggesting that NP and/or its secondary products were transferred to the edible portion of the lettuce. Dumont et al. (2022) used μ-PIXE to detect Cu accumulation in aquatic plant-myriophyllum spicatum exposed to CuO-NPs and Cu salts. They found that Cu salts were more toxic than similar accumulations, and this difference may be due to the rapid release of Cu^2+^ from Cu salts which that can lead to acute toxins.

In contrast to electron microscopy, fluorescence, or mass spectrometry imaging, the most important feature of synchrotron imaging is that it can be used to study the valence state of elements as well as their interactions with surrounding substances. After entering the plant, nanomaterials may go through complex processes before being internalized and utilized by the plant. Synchrotron radiation can thus be used to investigate the metabolic processes and ultimate fate of nanomaterials in plants.

As we know, many biological phenomena happen at multiple scales that may impose feedback on each other. Here we listed a table comparing different imaging methods in several aspects, including sample preparation, operation environment and lateral resolution (Table 1).

**TABLE 1:** Comparing of imaging tools that can be used to study interactions of nanomaterials with plants.

		Sample requirement and preparation		Operation environment	Lateral resolution	References
Electron microscopy	TEM	1. Tissue section and dry sample. 2. Coating with thin metal layer (usually gold) if sample is non-conductive or poor-conductive 3. Staining is unnecessary when preparing nanomaterial suspension.		Vacuum environment	∼0.07 nm	(Faisal et al., 2013; Zhang et al., 2022a; Zhao and Liu, 2022)
	SEM				∼0.8 nm	(Titze and Genoud, 2016; Li et al., 2020)
Optical microscope	CLSM	1. Living specimens can be observed. 2. No coating. 3. Non-fluorescent materials must be stained.		Ambient environment	∼140 nm	(Zhang et al., 2022a; Yan et al., 2022)
	TPM					(Helmchen and Denk, 2005; Bonilla-Bird et al., 2018)
	SRM				20–120 nm	(Von Wangenheim et al., 2016; Rillig et al., 2017)
	CRM	1. Tissue section if needed. 2. No coating	Living specimens can be observed		∼200 nm	(Schenzel et al., 2005; He et al., 2020)
	IRM		dry sample			(Schenzel et al., 2005; Chen et al., 2015)
Mass Spectrometry Imaging	LA-ICP-MS	1. Tissue section if needed			≥10 μm	(Chacón-Madrid and Arruda, 2018; Wojcieszek et al., 2019)
	SIMS	1. Tissue section if needed, dry sample and application of matrix if needed. 2. Labeling is unnecessary		Vacuum environment	50 nm-5 μm	(Aubert et al., 2012; Sekine et al., 2017)
	MALDI				10–200 μm	(Shiono et al., 2017; Liu et al., 2021a)
Synchrotron Radiation Techniques	μ-XRF				∼50 nm	(Hernandez-Viezcas et al., 2013; Lv et al., 2015)
	XAS					
	μ-PIXE				∼5 μm	(Larue et al., 2012; Dumont et al., 2022)

## 3 Monitoring of nanomaterial penetration and migration in specific tissues and their accumulation between different organs in whole plants

Like the animal body, the physiological environment of different plant organs is very different, which will directly affect the uptake, transport and accumulation of nanomaterials. By monitoring the penetration and migration of nanomaterials in various plant organs, researchers can have a more comprehensive understanding of nanoparticle induced plant toxicology, and help develop nanocarriers for various cargo delivery to specific plant organs with high efficacy Table 2.

**TABLE 2:** Summarization of nanoparticles that have been studied in plants.

NPs	Size	Coating	Morphology	Tool	Interaction compartments	Localization	References
Au NPs	5–20 nm (AuNS), 13 nm × 68 nm (AuNR)	DNA	Sphere, Rods	TEM	Leaf, cell walls	Outside cell (AuNS) or cytoplasm (AuNR)	Zhang et al. (2022b)
	15–40 nm	Polyethyleneimine, RNA	Cluster	CLSM	Leaf, cytoplasm	Cytoplasm	(Zhang et al., 2021; Zhang et al., 2022a)
SiO_2_ NPs	54 ± 7 nm	Bare	Particle	TEM	Leaf, cell walls	Outside cell	El-Shetehy et al. (2021)
CNTs	1 nm × 500 nm	Polyethyleneimine, RNA	Tubes	TEM	Cell	Cytoplasm and nucleus	Demirer et al. (2020)
	1 nm × 100 nm	Polyethyleneimine, pDNA,		CLSM	Cell	Cytoplasm and nucleus	Demirer et al. (2019b)
	1 nm × 120 nm	Chitosan, pDNA		CLSM	Cell	Chloroplasts	Kwak et al. (2019)
	3.2 nm × 200–1,000 nm	Polymer, peptides, pDNA		CLSM	Cell	Mitochondria	Law et al. (2022)
Cu-CNFs	95 nm × 230 nm	Bare	Tubes	SEM	Root, shoot and leaf	Outside cell	Ashfaq et al. (2017)
Polystyrene and polymethylmethacrylate particles	200 nm	Bare	Particle	SEM	Root, stem and leaf	Outside cell	Li et al. (2020))
Quantum dot	24.5 ± 2.5 nm	Peptides, β-cyclodextrin	Sphere	CLSM	Leaf, cell	Chloroplasts	Santana et al. (2020)
MWCNT, TiO_2_ NPs and CeO_2_ NPs	110–170 nm × 9 μm, 70–100 nm	Bare	Tubes, Particle	TPM	Root, shoot and leaf	Outside cell	Wild and Jones, (2009)
Cellulose synthase particles	25 nm	Bare	Rosette-shaped	SRM	Cell	Outside cell	Duncombe et al. (2022)
Ag NPs	10–40 nm	Bare	Particle	LA-ICP-MS	Leaf	Outside cell (40 nm Ag NPs) or cytoplasm (10 nm Ag NPs)	Chacón-Madrid and Arruda, (2018)
	10 nm, 60 nm	Polyethyleneimine, tannic acid	Particle	SIMS	Cell	Outside cell (60 nm Ag NPs) or cytoplasm (10 nm Ag NPs)	Sekine et al. (2017)
La_2_O_3_ NPs	20–30 nm	Bare	Particle	LA-ICP-MS	Leaf, stem	Intercellular spaces	Neves et al. (2019)
Mo NPs	550 ± 180 nm, 2.3 ± 0.5 μm	Bare	Particle	SIMS	Root	Outside cell	Aubert et al. (2012)
CeO_2_ NPs, ZnO NPs	8 nm, 10 nm	Bare	Particle	μ-XRF/XAS	Pod, stem, and nodule	Pod, stem, and nodule	Hernandez-Viezcas et al. (2013)
CuO NPs	40 nm	Bare	Particle	μ-XRF/XAS	Roots, lateral roots, and leaf	Outside cell	Peng et al. (2015)
	64.9 ± 8.5 nm	Bare	Cluster	μ-PIXE	Leaf	Outside cell	Dumont et al. (2022)
	50–1,000 nm	Bare	Particle	TPM	Root	Outside cell	Bonilla-Bird et al. (2018)
TiO_2_ NPs	14–665 nm	Bare	Particle	μ-PIXE	Leaf, root parenchyma and stele	Outside cell (>36 nm TiO2 NPs) or cytoplasm (<36 nm TiO2 NPs)	Larue et al. (2012)

### 3.1 Positron emission tomography

PET imaging technology, as a non-invasive medical and research tool, usually labelled biologically active molecules with a radioisotope, and realizes 3D imaging *via* collecting gamma rays emitted from the radioisotope. PET imaging has been widely used in pathological studies (e.g., tumors, psychiatry) for tracking the transport of drugs and nanomaterials in animals, and has be expanded into plant study related to nutrient, metabolite transportation (Garbout et al., 2012; Kanno et al., 2012; De Schepper et al., 2013; Karve et al., 2015; Hidaka et al., 2019; Hubeau et al., 2019).

Based on PET *in vivo* imaging and high biological tissue penetration depth. Therefore, by isotopic labeling of nanomaterials obtained during or after synthesis can be used to track the metabolism and tissue distribution of nanoparticles in living plants throughout the body (Liang et al., 2011, Figure 2H).

In order to understand the operation of element C in plants and the metabolic process of photo-assimilation, PET was utilized to detect and visualize the uptake of CO_2_ by plants, where CO_2_ is labeled with the short-lived radioisotope ^11^C (Garbout et al., 2012). In the mean time, the root structure of the plant was achieved through computed tomography scan (CT) imaging. Results showed that the concentration of ^11^C was highest in the main root of the radish (Kawachi et al., 2011), followed by the leaves. Results demonstrated that most of the metabolic activity of carbon occurred in the main root, indicating the principal pathway for the transport of photoassimilates was in roots. PET imaging for plant nanobiotechnology research is still under process, we have listed some relevant studies that may be useful for future research. PET imaging was used to track and quantify the transport and accumulation of copper-64-labeled nanoparticles (^64^Cu-NPs) in lettuce seedlings (Davis et al., 2017), and results showed that ^64^Cu-NPs were stable over the time range of 0.25–24 h. The transport of ^64^Cu-NPs into lettuce was not concentration-dependent but size-dependent. Radioisotope cerium-141 labelled ceramics nanoparticles (Ce NPs, 7 and 25 nm) were employed to incubate with cucumber plant leaves for 14 days, and the distribution of Ce NPs was traced *via* autoradiography (Zhang et al., 2011). Results showed that the distribution of Ce NPs of 7 nm and 25 nm was similar in cucumber leaves. Ce NPs accumulated only at the margin of young leaves, but widely distributed in older leaves, with lower concentrations in veins and petioles than other regions. It is suggested that the various physiological environments of old and young leaves might affect the migration of nanomaterials. In a similar study, the uptake and translocation of ^139^Ce-labeled CeO_2_ in plants were investigated (Schymura et al., 2017). Radiographic autoradiographic images of sunflower leaves exposed to ^139^Ce-CeO_2_ NPs revealed that NPs distribution was time-dependent. NPs accumulating primarily in leaf veins and margins at the end of the transport water flow for a short period of time, whereas for a longer period of time (1 week), NPs were uniformly distributed throughout the plant, possibly due to dissolution of CeO_2_ NPs then transportation as ions in plants. Although autoradiography is easier to obtain, only two-dimensional images can be obtained *via* this method and the resolution is lower. However, PET imaging can obtain three-dimensional pictures of the sample. When extension to nanomaterials, it is more convenient to use PET or single-photon emission computed tomography (SPECT) to track the transport of radioisotope labelled nanomaterials in plants. Isotope can be labelled inside the nanomaterial instead of on the surface, which provides similar surface and chemical composition of the labeled and unlabeled nanomaterials. Moreover, the labeled radioisotopes can be avoided from detaching from the nanomaterial, which are inevitable in fluorescent labeling mediated imaging methods.

Despite the benefits of PET imaging with high sensitivity, specificity and entire body imaging, there are still issues for PET imaging in plants, such as the need for radiolabeling of nanomaterials and the high cost of the specialized instruments.

### 3.2 Magnetic resonance imaging

MRI uses the principle of nuclear magnetic resonance to perform imaging. By applying a high-intensity magnetic field, intrinsic magnetic moment of the material is rearranged and converted to a high-energy state, when it returns to a low-energy state, radio frequency is released as an NMR signal for imaging.

Till now, there are very few studies in plant nanobiotechnology using MRI, and the authors found only one example. Huang et al. (2011) modified cubic iron oxide NPs with carboxyl-terminated PEGylated phospholipids (HOOC-PEG-PL) and assembled Brome mosaic virus (BMV) capsid proteins (CPs) on the surface to obtain virus-based nanoparticles (VNPs). The initial idea was to track the transport of VNPs in the plant vasculature system by MRI and study the migration of protein-based macromolecules using VNPs as a model. However, they just injected cubic iron oxide NPs coated with HOOC-PEG-PL into the abaxial side of *N. benthamiana* leaves *via* a needle-free injection method and used MRI to observe the entire leaf to examine the potential of NPs as a contrast agent for plant MRI studies.

MRI allows deep 3D imaging of living plants, but what distinguishes MRI with PET is that MRI can simultaneously image the internal structure of the plant itself, which allowing for the ability to co-locate nanomaterials with specific plant tissue sites.

### 3.3 X-ray computed tomography

CT is an imaging technique that has advantages in non-destructive testing, simple sample preparation and whole-body 3D imaging *in vivo*. The principle of CT is that X-rays emitted from the X-ray source are absorbed or dispersed as passing through the specimen, high density materials and high atomic number elements tend to produce higher attenuation and hence produce different contrasts for imaging. The use of X-ray computed tomography to track the migration and distribution of nanomaterials in animal tissues has been studied in the past, and has also been extended for plant organ and tissue imaging (Piovesan et al., 2021). However, so far, there is no application of X-ray CT to monitor the transport process of nanomaterials in plants. But some examples of using CT to track nanomaterial migration in animals can be instructive for future plant researchers. Synthetic polymer-coated Bi_2_S_3_ nanoparticles (BPNPs) were injected into mice through the tail vein, and then used CT to track the migration of BPNPs in mice (Rabin et al., 2006). Results indicated that BPNPs were primarily present in the ventricle and all major arterial and venous structures initially, with a circulating half-life of 140 ± 15 min. The BPNPs were distributed to phagocytic-containing organs (liver, spleen, lymph nodes) after 12–24 h, some were distributed in the kidneys and urinary tract after 1–2 days, which may be related to renal shedding. In addition, they also verified that BPNPs can localize to lymph nodes.

Similar to MIR, CT has been widely used in the past for 3D imaging of plant organs and tissues (Piovesan et al., 2021). Therefore, CT can be used to monitor the transport of nanoparticles in specific plant tissues by simultaneously imaging nanomaterials and plant tissues. It is worth noting that NPs must have good CT contrast for it to be tracked in plants by CT, otherwise they may be obscured by the background of the plants.

## 4 Other technologies and conclusion

Other techniques may also hopefully be extended to plant nanotechnology to determine the uptake of nanoparticles by plants. Khodakovskaya et al. (2011) developed a photothermal and photoacoustic cell scanning platform to observe MWCNTs in tomato plants. It used CNTs to absorb laser light to generate photoacoustic signals for detection. Through this technique, they detected carbon nanotubes in leaves and tomato fruit. Atomic force microscopy (AFM) has the characteristics of high-resolution and non-destructive imaging, which is very useful for studying the interaction between nanomaterials and plant cell surfaces, and may be used to quantify the mechanical parameters of the interaction between nanomaterials and plant cells. AFM silicon nitride tips were modified with hydrophobic (oleylamine coated Cu_2-x_Se NPs (CS@OA)) and hydrophilic (chitosan coated Cu_2-x_Se NPs (CS@CH) particles (Sharma et al. (2020). Using a microscope, the AFM tip was contacted with the root tip to study the interaction force between them, and the results revealed that CS@OA NPs had a stronger interaction with the root, indicating the maximum adhesion force expressed by the hydrophobic NPs, resulting in increased root uptake. Infrared spectroscopy (IR) (including near-field scanning optical microscope, photothermal microspectroscopy, nanoscale fourier transform infrared spectroscopy, atomic force microscope based infrared spectroscopy) is used to identify chemical substances or functional groups in solid, liquid or gaseous form by measuring the interaction of infrared radiation with matter through absorption, emission or reflection. It can be used to characterize new materials or to identify and validate known and unknown samples. Thus, IR spectroscopy microscopy is also a potential promising characterization tool for plant nanotechnology.

## 5 Conclusion and perspectives

In conclusion, it has been shown that different imaging techniques can detect and quantify the absorption, transportation and accumulation of nanomaterials in plants with different penetration ability, various time and spatial resolution. These techniques include microscopy (optical, scanning and electron microscopy) and spectroscopic techniques (atomic spectroscopy, synchrotron radiation, particle-induced X-ray emission, Raman spectroscopy, infrared microscopy and photothermal/photoacoustic techniques). The development of nanomaterials with organ-tissue-cell-organelle targeting capability is of specific importance for practical applications because it can be used for efficient targeted delivery of pesticides, fertilizers, nucleic acids, proteins, and other substances for green and efficient crop growth, disease control, genetic modification, and other purposes. In order to realize these visions, it is important to characterize the manufactured nanomaterials with suitable tools for further practical applications.

Starting from the spatial scale, PET/MIR/CT are able to scan the whole body of plants and determine the distribution of nanomaterials within tissues and organs, which help analyze the metabolism/migration/accumulation process of nanomaterials in plants. Once localized in a specific organ, in-depth imaging methods with better resolution are required to see the location of nanomaterials inside cells and the interaction with subcellular organelles. Specifically, label-free techniques like LA-ICP-MS/MALDI/IRM/CRM can be utilized to observe the distribution of nanomaterials in certain tissues or organs; SIMS and fluorescence microscopy (TPM/CLSM/SRM) can be used to study the distribution of nanomaterials at the plant cell-subcellular level in order to better understand their cell penetration and subcellular localizations. In addition, real-time imaging capabilities of fluorescence microscopy is suitable to observe how nanomaterials interact with plant cells and subcellular structures. Synchrotron radiation (μ-PIXE/XAS/μ-XRF) can also be used to image the distribution of nanomaterials in tissue and cell level, and notably, this approach is able to map the elements within the surrounding medium, which can help to study the internalization pathways and final fate of nanomaterials in plants. High-resolution feature of electron microscopies (SEM, TEM) gives them the ability to observe nanomaterial distribution in plant subcellular organelles for further subcellular targeting ability evaluation or associated kinetic research. Similarly, AFM can map the distribution of nanomaterials on cell surfaces. Needle tip control can also be used to probe the mechanical parameters of nanomaterials and plant cell surfaces to study the interaction between nanomaterials with different properties and plant cells.

Another thing that needs to be considered is that some nanomaterials may have short or long term effects after entering plant cells. Some metal and their composite nanomaterials (quantum dots, etc.) were reported to inhibit DNA replication, causing oxidative stress, damaging root structure, and impede photosynthesis in plants (Dev et al., 2018; Zhu et al., 2019). These negative effects eventually led to stunted growth and promote senescence and death of the plant. When exposed to Ag_2_S NPs, no effect was seen during a short-term period of 24 h, but long-term exposure over 2 weeks reduced wheat growth by up to 52% (Wang et al., 2015). Polystyrene nanoplastics (PSNPs) are found to absorb onto roots and transferred to sprouts, affecting root cell ultrastructure, germination process, seedling growth, and root mitotic activity, thus inducing cytogenetic aberrations (Spanò et al., 2022). In contrast, nanomaterials with enzyme-like catalytic activity (nanozymes) can improve photosynthetic efficiency and stress resistance of plants by removing excess reactive oxygen species (ROS) in plant cells (Zhao et al., 2022). Therefore, how nanomaterials affect plants depends on their chemical composite and intrinsic properties, giving the chance for researchers to choose and design nanomaterials for their study.

Of note, different methods require corresponding sample preparation procedure, complex data analysis process. Researcher have been utilizing two or more imaging techniques (such as TEM or SEM plus XRF) to obtain multi-dimensional information in plant nanotechnology (Lv et al., 2015; Liu et al., 2019). Thus, in the actual research process, appropriate selection of multiple imaging tools not only enables subcellular localization of nanomaterials at different spatial scales (Table 2), but also benefits further studies on the kinetic mechanisms of nanomaterial-plant interactions, as well as the metabolic pathways and fate of nanomaterials analysis in plants. Therefore, the author suggests that employing a combination of several techniques can provide a more comprehensive understanding of the interactions between nanomaterials and plants, from microscale to macroscale and microscale.
